# Clinicopathological features of choledocholithiasis patients with high aminotransferase levels without cholangitis: Prospective comparative study: Erratum

**DOI:** 10.1097/MD.0000000000005661

**Published:** 2016-12-09

In the article “Clinicopathological features of choledocholithiasis patients with high aminotransferase levels without cholangitis: Prospective comparative study”,^[1]^ which appeared in Volume 95, Issue 42 of *Medicine*, Dr. Joon Seong Park's name was originally misspelled.
